# Genetic variation in 117 myelination-related genes in schizophrenia: Replication of association to lipid biosynthesis genes

**DOI:** 10.1038/s41598-018-25280-4

**Published:** 2018-05-02

**Authors:** Tomasz Stokowy, Tatiana Polushina, Ida E. Sønderby, Robert Karlsson, Sudheer Giddaluru, Stephanie Le Hellard, Sarah E. Bergen, Patrick F. Sullivan, Ole A. Andreassen, Srdjan Djurovic, Christina M. Hultman, Vidar M. Steen

**Affiliations:** 1 NORMENT – KG Jebsen Centre for Psychosis Research, Department of Clinical Science, University of Bergen, Bergen, Norway; 2 Dr. Einar Martens Research Group for Biological Psychiatry, Center for Medical Genetics and Molecular Medicine, Haukeland University Hospital, Bergen, Norway; 3 Department of Medical Genetics, Oslo University Hospital, Oslo, Norway; 4 NORMENT – KG Jebsen Centre for Psychosis Research, Department of Clinical Medicine, University of Oslo, Oslo, Norway; 5 Department of Medical Epidemiology and Biostatistics, Karolinska Institutet, Stockholm, Sweden; 6 Center for Psychiatric Genomics, Department of Genetics, University of North Carolina, Carolina, USA; 7 NORMENT – KG Jebsen Centre for Psychosis Research, Division of Mental Health and Addiction, Oslo University Hospital, Oslo, Norway

## Abstract

Schizophrenia is a serious psychotic disorder with high heritability. Several common genetic variants, rare copy number variants and ultra-rare gene-disrupting mutations have been linked to disease susceptibility, but there is still a large gap between the estimated and explained heritability. Since several studies have indicated brain myelination abnormalities in schizophrenia, we aimed to examine whether variants in myelination-related genes could be associated with risk for schizophrenia. We established a set of 117 myelination genes by database searches and manual curation. We used a combination of GWAS (SCZ_N = 35,476; CTRL_N = 46,839), exome chip (SCZ_N = 269; CTRL_N = 336) and exome sequencing data (SCZ_N = 2,527; CTRL_N = 2,536) from schizophrenia cases and healthy controls to examine common and rare variants. We found that a subset of lipid-related genes was nominally associated with schizophrenia (p = 0.037), but this signal did not survive multiple testing correction (FWER = 0.16) and was mainly driven by the *SREBF1* and *SREBF2* genes that have already been linked to schizophrenia. Further analysis demonstrated that the lowest nominal p-values were p = 0.0018 for a single common variant (rs8539) and p = 0.012 for burden of rare variants (LRP1 gene), but none of them survived multiple testing correction. Our findings suggest that variation in myelination-related genes is not a major risk factor for schizophrenia.

## Introduction

Schizophrenia (SCZ) is a serious psychotic disorder with estimated high heritability (0.64–0.81)^1–3^. For a long time, the nature of this genetic susceptibility was not known, but this situation has changed markedly during the last 10 years, especially due to the success of the Psychiatric Genetics Consortium (PGC). So far, 108 gene loci have been significantly associated with SCZ by genome-wide association studies (GWAS)^4^. The HLA region on chromosome 6 is by far the most significant, including the complement factor 4 locus^5^. Those common variants account for an estimated 23% of the total heritability, but each variant has only minor influence on the disease risk, with odds ratios typically below 1.10^3,4^. Copy number variants (CNVs) constitute another major type of genetic susceptibility. Such CNVs are rare and may be recurrent (e.g., loci on 1q21.1, 2p16.3 (*NRXN1*), 3q29, 7q11.2, 15q13.3, 16p11.2 proximal, 16p11.2 distal, and 22q11.2), usually leading to markedly increased risk of schizophrenia in addition to several neuropsychiatric conditions^6–9^. Finally, it has been shown that schizophrenia is also associated with inherited, ultra-rare gene-disrupting and putatively protein-damaging variants, mainly in neuron-expressed genes that encode synaptic proteins^10^.

Despite these important findings, there is still a large gap between the estimated and explained heritability. Alternative approaches are therefore needed to identify more of the genetic factors, and one possibility is to focus on hypotheses driven by the pathophysiology of schizophrenia. Several structural MRI studies including diffusion tensor imaging (DTI) have observed reduced white matter volume and changes in the fibre tracts in patients with schizophrenia^11–13^. Other studies have demonstrated reduced expression of myelination-related genes in post-mortem brain samples^14–16^. These data suggest that myelination abnormalities could be implicated in schizophrenia. Myelin is a multi-lamellar structure that wraps around the axons of the neurons. In the central nervous system (CNS), it is formed by the lipid-rich membrane of oligodendrocytes. The myelination process is complex and temporal, involving specification and proliferation of oligodendrocyte precursor cells (OPCs), differentiation of mature oligodendrocytes, with subsequent synthesis and turnover of structural myelin components including specialized proteins and lipids^17,18^. Previously, genes implicated in glial functions (astrocytes and oligodendrocytes) have been associated with schizophrenia when tested in gene enrichment analyses^19–24^. The aim of this study was to test more specifically the implication of genes related to myelination as a risk factor for schizophrenia, using a combination of common and rare variants in GWAS, exome chip and exome sequencing data.

## Material and Methods

### Generation of CNS myelin-related gene set

The CNS myelin-related gene set was generated in a two-step process. First, we queried the Gene Ontology (GO) Consortium database (geneontology.org) by the search terms “myelin” and “myelination”, which gave a large number of GO terms, including “myelination” (436 gene products), “myelin sheath” (214 gene products), “regulation of myelination” (73 gene products), and “central nervous system myelination” (67 gene products). Due to the hierarchical tree structure of the GO database, more specific GO terms with fewer genes were in general contained within the broader terms “myelination” and “myelin sheath”. Since the GO databases may be biased by research focus, and may include both type I and type II errors^25,26^, the different gene lists were manually curated to select genes with an biologically well-established link to myelin and myelination in the CNS, by careful review of the literature, similar to the approach of Devor *et al*.^27^. This enables exclusion of genes with weak documentation of the annotation or functional link to myelin in the peripheral nervous system only. In addition, we included a selection of genes that are involved in lipid biosynthesis and transport (due to the role of lipids in myelination) or in monogenic disorders with structural myelin defects.

The complete CNS myelin-related gene set included a total of 117 genes - 113 protein-coding genes, 2 miRNA, 1 lincRNA and 1 antisense gene (see Supplementary File 1), which was subdivided into genes with biologically known involvement in (1) oligodendrocyte precursor cell (OPC) specification and proliferation (N = 13 genes), (2) oligodendrocyte differentiation and myelination (N = 49 genes; including structural CNS myelin proteins), (3) lipid biosynthesis and transport (N = 29 genes) and (4) various other genes involved in myelination and myelin-related disorders (N = 26 genes).

### Clinical samples

The CNS myelin-related gene set was queried in three different samples: (1) a multicenter GWAS sample of 35,476 schizophrenia cases and 46,839 controls from the Psychiatric Genetics Consortium (PGC SCZ2), (2) a Norwegian sample of 269 schizophrenia cases and 336 controls genotyped by the Illumina Exome chip, and (3) a Swedish sample of 2,527 schizophrenia cases and 2,536 controls that were exome sequenced (completely overlapping part of PGC SCZ2 for which we have access to raw data).

#### PGC schizophrenia GWAS samples

Summary statistics from the PGC-SCZ GWAS were downloaded from https://www.med.unc.edu/pgc/results-and-downloads. The data consist of summary statistics of 9,444,231 imputed variants, on 35,476 cases and 46,839 controls from 46 cohorts of European ancestry (PGC SCZ2 discovery set)^4^. SNPs with poor imputation (imputation score < 0.9) or minor allele frequencies < 0.1, ambiguous SNPs, and insertions/deletions were filtered out, leaving a total of 3,511,943 markers for analysis.

#### Norwegian exome chip-genotyped schizophrenia sample

The Norwegian sample consisted of 269 schizophrenia cases and 336 controls (605 unrelated individuals in total) from the TOP study^28^. All samples had been genotyped with the Illumina HumanExome Beadchip v1.0 array and re-analyzed from raw data using Illumina Genome Studio, according to the best practice from the CHARGE consortium^29^. Quality of called variants was improved using zCall^30^. Hardy-Weinberg equilibrium statistics of variants were tested by PLINK^31^. PLINK/SEQ was applied to transform accepted variants into a vcf file. 1,536 variants (MAF > 0) located within 117 myelination genes were extracted using VCFtools^32^. In 12 of these variants, the alternative allele was more frequent than the reference allele, so these variants were transposed to match hg19 reference.

#### Swedish exome sequenced schizophrenia sample

The Swedish sample consisted of 2,527 schizophrenia cases and 2,536 controls (5,063 unrelated individuals in total). All samples were sequenced using either the Agilent SureSelect Human All Exon Kit (29 Mb) or the Agilent SureSelect Human All Exon v.2 Kit (33 Mb) for exome capture^33^. Sequencing was performed by Illumina GAII or Illumina HiSeq2000. Sequence data were aligned and variants called by the Picard/BWA/GATK pipeline^34^. On the basis of Exome Chip data and validation of selected variants by Sanger sequencing, a high sensitivity and specificity of singleton calls was estimated as earlier described^33^. Variant frequencies are available in the dbGaP study phs000473.v1 (http://research.mssm.edu/statgen/sweden). 4,766 variants located within 117 RefSeq defined myelination genes were extracted using VCFtools^32^. In 46 of these variants, the alternative allele was more frequent than the reference allele, so these variants were transposed to match hg19 reference.

### Gene Set Enrichment Analysis (GSEA) in GWAS data

All SNPs in the PGC-SCZ GWAS data were annotated to known NCBI RefSeq genes (date: August 1, 2016)^35^. The list contained 27,063 genes; in case of several variants, the longest was selected. Since the LD structure of the major histocompatibility complex (MHC) region is challenging, and genes from this region are overrepresented in SCZ GWAS, we excluded genes from hg19 chr6:25–34 Mb, with 26,730 genes remaining. SNPs were assigned to genes if they were a) located within the boundaries of the gene+/−10 kb upstream and downstream or if b) in LD (r^2^ ≥ 0.20) with another SNP located within the boundaries of the gene (+/−10 kb) using the LDsnpR tool^36^. For LD estimation, we used the European cohort from the 1000 Genomes Project^37^.

For each gene, a combined p-value score was calculated according to the Brown method^38,39^. The technique allows generation of combined p-value scores for dependent tests, where the correction procedure is based on the LD structure within the structural bin. The gene scores were log transformed and ranked in decreasing order.

The Gene Set Enrichment Analysis (GSEA)^40^ method was developed for global gene expression studies. However, this technique has also been used for GWAS studies^41,42^. The GSEA performs running sum statistics and calculates an enrichment score for each of the selected myelin-related genes, considering their rank in the pre-ranked gene list from the SCZ GWAS. The significance of the score was calculated using 1,500 permutations of the ranked list.

### Analysis of common and rare variants in the exome sequence

Common variants (defined as minor allelic frequency > 0.1) in the 117 myelin-related genes were subject to case control association analysis, using all variants extracted from either the exome chip (Norwegian sample) and exome sequence (Swedish sample) data, as described above. Tests of association were performed using EPACTS single variant Logistic Score Test^43^, in accordance with the tool documentation.

Rare variants (defined as minor allelic frequency ≤ 0.1) extracted from the Norwegian and Swedish datasets were first analyzed for their potential differential clustering between cases and controls within certain genes (i.e. gene burden analysis), using the SKAT-O test^44^ according to the EPACTS tool documentation.

We also examined the total number of loci with rare variants per individual (both heterozygous and homozygous, investigated separately) in the total set of 117 genes and the four subsets of genes in the Norwegian and Swedish samples. This analysis method is an approach to extend SKAT-O, which tests variant burden per gene. In contrast to SKAT-O, we test the burden of variants in groups of genes that are related to a certain function. The analysis was performed by reading vcf files into the R/Bioconductor environment, using the VariantAnnotation package^45^. Comparison of median values of variant counts were used to avoid influence of possible outliers. The difference in variant load between cases and controls was tested by Welch t-test.

Finally, loss of function (LoF) variants in the 117 myelin-related genes in the Swedish sample were annotated and selected using snpEff^46^ with -lof parameter. Their distribution in cases versus controls was tested with chi2 test in R. We also counted the total number of LoF singleton variants and compared their distribution in cases versus controls.

## Results

### Lipid biosynthesis genes are associated to schizophrenia in schizophrenia GWAS data

As a first step, we sought to investigate the association of myelin gene groups with schizophrenia in the largest schizophrenia GWAS dataset to date, PGC. The lowest p values for the myelination genes are provided in Supplementary File 1, with the most significant finding being p = 1.806 * 10^−13^ for rs115370182 (an intron variant in the MOG gene).

By using the publicly available PGC SCZ2 data, we performed a gene set enrichment analysis (GSEA) on the total myelination gene set (117 genes) as well as on the four subsets of genes (see Table 1). There was no significant enrichment for the myelination genes in total. One of the four subsets, the lipid biosynthesis and transport-related genes, demonstrated nominally significant enrichment of association to schizophrenia (p = 0.037), but this signal did not survive correction for multiple testing (FWER = 0.16). Moreover, further analysis of the subset revealed that *SREBF1* and *SREBF2* were ranked as no. 1 and 2 among the leading edge genes (Supplementary Table 1). Since both genes have previously been associated with schizophrenia^47^, the subgroup analysis was re-run after exclusion of *SREBF1* and *SREBF2*. The remaining subset of 27 genes failed to display any significant association (p = 0.32). No enrichment of association with schizophrenia was found for the three other gene subsets “OPC specification and proliferation”, “oligodendrocyte differentiation and myelination”, and “other genes involved in myelination and myelin-related disorders” (Table 1).

**Table 1:** Gene set enrichment analysis (GSEA) for myelin gene sets.

Name of gene set	No. of genes	ES	NES	NOM p-value	FDR q-value	FWER p-value
Myelination genes (total gene set)	117	0.49	1.16	0.072	0.072	0.072
Oligodendrocyte precursor cell (OPC) specification and proliferation (subset 1)	13	0.42	0.85	0.721	0.766	0.997
Oligodendrocyte differentiation and myelination (subset 2)	48	0.48	1.09	0.309	0.441	0.779
Lipid (incl. cholesterol) biosynthesis and transport (subset 3)	29	0.62	1.37	**0.037**	0.165	0.156
Other genes involved in myelination and myelin-related disorders (subset 4)	26	0.53	1.16	0.234	0.443	0.627

The GSEA performs running sum statistics and calculates an enrichment score for each of the selected myelin-related genes, considering their rank in the pre-ranked gene list from the SCZ GWAS. The significance of the score was calculated using 1,500 permutations of the ranked list. Abbreviations: ES – enrichment score; NES – normalized enrichment score; NOM – nominal p-value; FDR – false discovery rate; FWER – family-wise error rate.

### No association with schizophrenia for neither common nor rare variants within the coding sequence of the myelination genes

**Figure 1:**
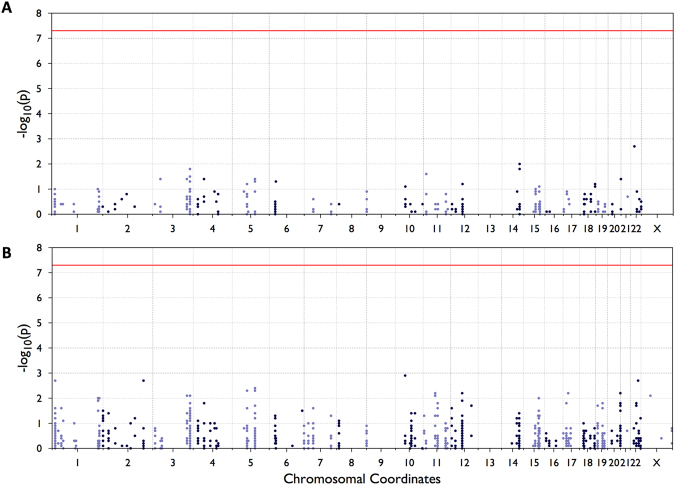
Manhattan plots for common (MAF > 0.05) variants in Norwegian (**A**) and Swedish (**B**) cohorts. Variants located in myelination genes do not reach genome-wide significance.

The next step was to examine association of common variants located in myelination genes to schizophrenia (analysis design provided in Supplementary File 2). We had access to a Norwegian sample of 269 schizophrenia cases and 336 controls (605 unrelated subjects in total) that had been genotyped with the Illumina Human Exome array, and a Swedish sample of 2,527 schizophrenia cases and 2,536 controls (5,063 unrelated individuals in total) that had been exome sequenced. We observed a total of 1,536 and 4,766 different variants within the 113 protein-coding and 4 non-coding myelination-related genes in the Norwegian and Swedish sample, respectively, including 185 and 187 common variants in the two samples (defined as MAF > 0.1). By logistic regression analysis of each common variant, we found no significant differences between cases and controls that survived correction for multiple testing (EPACTS, 5 * 10^−8^), with the lowest nominal p-value being 1.8 × 10^−3^ in Swedish data set (Fig. 1 and Table 2, Supplementary Files 3 and 4).

**Table 2:** Overview of the distribution of common and rare variants in myelination-related genes in schizophrenia patients and healthy controls.

	Norwegian Sample			Swedish Sample		
N, schizophrenia patients/healthy controls	269/336			2,527/2,536		
Genotyping platform	Illumina *HumanExome Beadchip* v1.0			Illumina Exome Sequencing		
SNPs and indels in myelination-related gene set	1,536 (1,532 with correct reference^4^, with reversed reference and variant allele)			4,766 (4,720 with correct reference^46^, with reversed reference and variant allele)		
Total gene set (variant counts per sample, median values)	Schizophrenia:	Control:	t-test p- value:	Schizophrenia:	Control:	t-test p- value:
- heterozygous	**50**	49	0.28	74	75	**0.03**
- homozygous alternative	11	11	0.85	20	20	0.87
Subset 1: Oligodendrocyte precursor cell specification and proliferation	Schizophrenia:	Control:	t-test p value:	Schizophrenia:	Control:	t-test p value:
- heterozygous	8	8	0.72	7	7	0.81
- homozygous alternative	2	2	0.78	1	2	0.6
Subset 2: Oligodendrocyte differentiation and myelination	Schizophrenia:	Control:	t-test p- value:	Schizophrenia:	Control:	t-test p- value:
- heterozygous	20	20	0.76	25	25	0.1
- homozygous alternative	4	5	0.79	9	9	0.58
Subset 3: Lipid (including cholesterol) biosynthesis and transport	Schizophrenia:	Control:	t-test p- value:	Schizophrenia:	Control:	t-test p- value:
- heterozygous	11	12	0.32	21	21	0.38
- homozygous alternative	3	3	0.71	5	5	0.77
Subset 4: Other genes involved in myelination and myelin-related disorders	Schizophrenia:	Control:	t-test p- value:	Schizophrenia:	Control:	t-test p- value:
- heterozygous	10	9	**0.007**	22	22	0.09
- homozygous alternative	2	2	0.84	4	4	0.77
Common variants tested (MAF > 0.1)	185			187		
Most significant common variants	p = 0,037			p = 0,0018		
Lowest p-value - Logistic Score	3:39523003_A/G_Intron:MOBP			2:198362018_T/C Synonymous: HSPD1		
Test - single variant association	AF SCZ: 0.76, AF CTR: 0.71			AF SCZ: 0.66 AF CTR:0.63		
Most significant rare variant gene	0,03			0,012		
Lowest p-value - SKAT-O Test - gene burden association for rare variants	chr3:184035172-184049550_EIF4G1			chr12:57532238-57606021_LRP1		
Loss of function (LOF) variants in the data set	6			99		
Loss of Function variants (not singletons), lowest p-value	Not evaluated (array technology/insufficient sample number for reliable analysis)			Schizophrenia:	Control:	chi2 p- value:
PADI2 chr1:17410253, heterozygous				7	18	**0.03**
NPC2 chr14:74947404 heterozygous				22	16	0.32
LOF singletons (present in one individual only)	Not evaluated (array technology/insufficient sample number for reliable analysis)			Schizophrenia:	Control:	Chi square test:
				38	33	p = 0.38

Table summarizes variant load per subject, single variant association, gene burden association and loss of function variant analysis. T-test and chi2 test values were computed in R. Significant differences are marked with bold font, however they did not survive correction for multiple testing (q > 0.05).

To test for accumulation of rare variants within a gene, we performed a gene burden analysis with SKAT-O. Differences between cases and controls in the Norwegian (lowest p-value 0.03; *EIF4G*^44^) and Swedish samples (lowest p-value 0.012; *LPR1*) (Table 2) did not survive correction for multiple testing. Detailed information of the SKAT-O gene burden analysis is provided in Supplementary Files 5 and 6.

**Figure 2:**
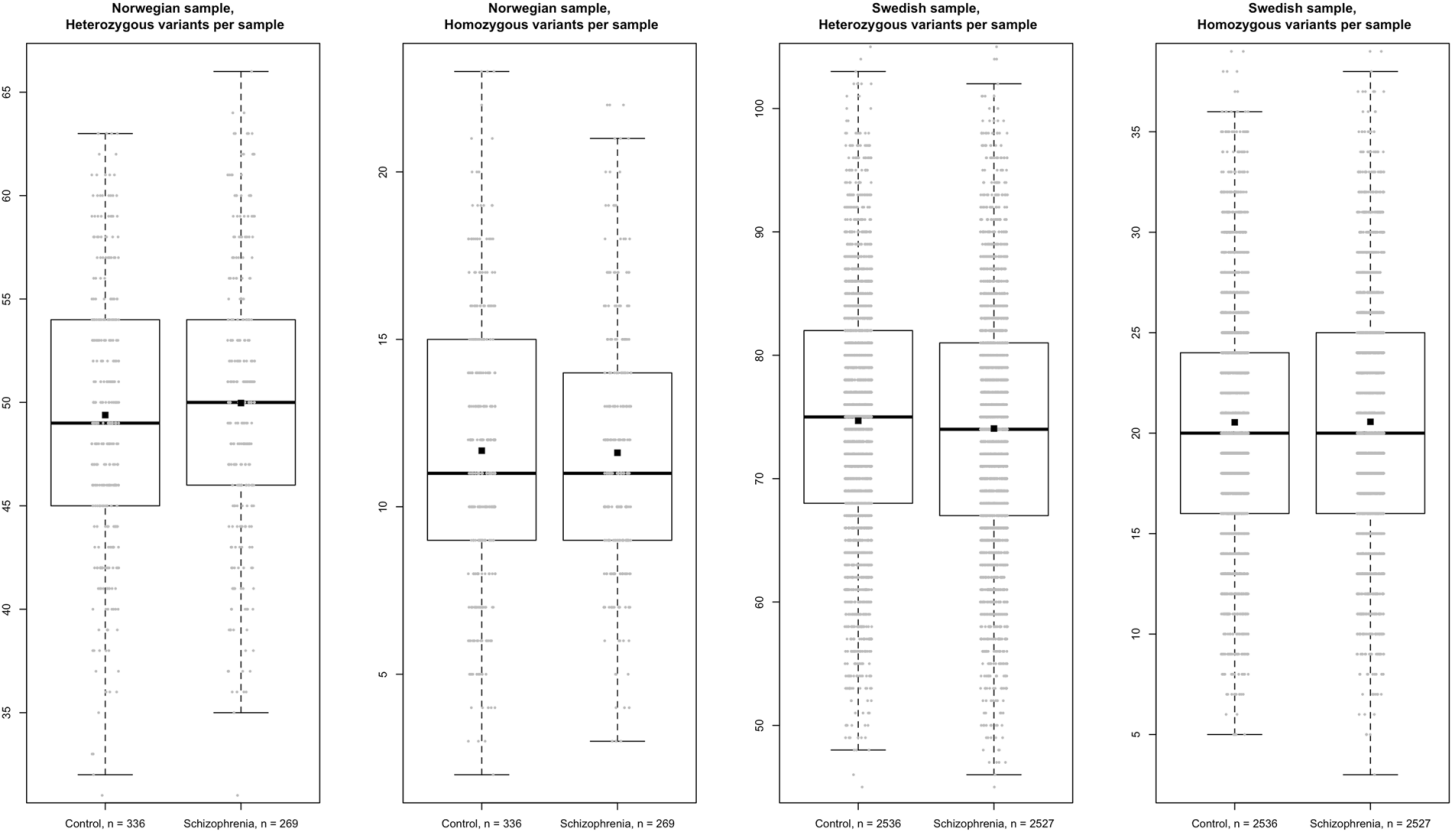
Distributions of heterozygous and homozygous alternative variant counts in Norwegian and Swedish samples. Boxplots represent first quartile, median and third quartile. Mean values are additionally marked with squares. Each grey dot represents number of myelination gene variants in one subject.

In theory, a combination of variants in several myelination genes in the same subject could impose increased risk. We therefore examined the load of such variants in cases and controls with respect to the total gene set and each of the four subsets. We measured variant load by calculating cumulative number of heterozygous and homozygous variants. We observed a small difference between cases and controls in the median number of heterozygous variants per subject for all myelination genes in the Swedish sample, but the p-value was not significant after correction for multiple testing (74 in cases versus 75 in controls; t-test p = 0.03; q = 0.30; Fig. 2). For the subset of “other genes involved in myelination and myelin-related disorders” in the Norwegian sample (10 in cases versus 9 in controls; p = 0.007) (Table 2). These differences were minor and none of them were supported by merged data from both samples.

### No excess of loss-of-function variants in the myelination genes

In a final step of the analysis, we approached all loss-of-function (LoF) variants, defined as nonsense, frame shift (indels), splice gain and splice loss mutations. Due to a low number of such variants in the Norwegian sample (6 in total), we only used the Swedish dataset that contained 99 different LoF variants across all individuals, including 73 ultra-rare singletons (38 observed in the group of 2,527 schizophrenia patients and 33 among the 2,536 controls; p = 0.55) (Table 2). The remaining 28 non-singleton LoF variants were checked for differences between cases and controls, of which a heterozygous variant in *PADI2* (stop gain; allele frequency 0.0015 in gnomAD database) had the lowest p value (chi square test; OR = 0.37, p = 0.03), but the p-value did not survive correction for multiple testing (Table 2).

## Discussion

In the current study, we examined the role of common and rare variants in myelination-related genes as risk factors for schizophrenia, using a combination of GWAS, exome chip and exome sequencing data in different case control samples. We observed a nominally significant enrichment of common SNPs in a subset of lipogenesis-related genes in schizophrenia patients (p = 0.037), but conservative correction for multiple testing rejected this finding (FWER = 0.16). Moreover, the trend signal observed in this subset was mainly driven by variants in the well-known schizophrenia-associated genes *SREBF1* and *SREBF2*.

Indeed, *SREBF1* and *SREBF2*, encoding the SREBP1 and SREBP2 transcription factors that control fatty acid and cholesterol biosynthesis, were ranked as the two leading edge genes in the GSEA. Since *SREBF1* and *SREBF2* on chromosome 17p11.2 and 22q13.2 have both been associated with schizophrenia^4,48^, the GSEA analysis of the PGC2 schizophrenia GWAS data was re-run after exclusion of these genes. The remaining subset of 27 genes failed to display any significant findings, demonstrating that the signal of association was mainly driven by the *SREBF* gene loci. It remains to be clarified whether variants in *SREBF1* and *SREBF2* influence the risk of schizophrenia through effects on lipid biosynthesis and myelination in the brain.

There was no significant enrichment in the PGC2 schizophrenia GWAS data for the total set of myelination genes or for any of the three other subsets of genes, thereby excluding a major role of common SNPs in these genes as disease susceptibility factors. This result is in line with a study by Goudriaan *et al*.^19^ who reported an association between schizophrenia and lipid metabolism genes in the PGC1 GWAS data, but no significant findings for a set of 17 myelination genes.

We also examined coding variants in the myelination genes, using exome chip and exome sequencing data. In general, the analyses of single variants, gene burden effects (i.e. number of variants within one gene) and variant load per subject (i.e. accumulation of variants in several genes in one person) did not display any statistically significant associations that survived correction for multiple testing. It is therefore unlikely that coding variants in the myelination-related genes play any major role as risk factors for schizophrenia. This is supported by the recent finding that the increased burden of ultra-rare, gene-disruptive variants in schizophrenia is mostly related to variants in neuronally expressed genes that encode proteins with synaptic localization^10^. It should be noted that the Swedish exome sequence data used by us overlap with the latter report.

The main strength of our study is the combination of a hypothesis-driven approach, a manually curated set of myelination-related genes and the analysis of both common and rare variants in different samples. The main weakness is the limited statistical power to detect associations between rare variants and schizophrenia, thereby lowering the value of the negative findings.

In conclusion, we have confirmed that non-coding variants linked to the *SREBF1* and *SREBF2* genes are associated with increased risk of schizophrenia. Our data show that variants located within other myelination related genes are unlikely to be major susceptibility factors in schizophrenia.

## Electronic supplementary material

Supplementary Dataset 1

Supplementary File 2

Supplementary Dataset 3

Supplementary Dataset 4

Supplementary Dataset 5

Supplementary Dataset 6
