# Supplementary material for: Genetic variation in 117 myelination-related genes in schizophrenia: Replication of association to lipid biosynthesis genes
Source: Sci Rep. 2018 May 2;8:6915. doi: 10.1038/s41598-018-25280-4 (PMC5931982; doi:10.1038/s41598-018-25280-4)

Genetic variation in 117 myelination-related genes in schizophrenia: Replication of association to lipid biosynthesis genes

Supplementary Information

Tomasz Stokowy[1,2], Tatiana Polushina[1,2], Ida E. Sønderby[3,4], Robert Karlsson[5], Sudheer Giddaluru[1,2], Stephanie Le Hellard[1,2], Sarah E. Bergen[5], Patrick F. Sullivan[5,6], Ole A. Andreassen[4,7], Srdjan Djurovic[1,3], Christina M. Hultman[5] and Vidar M. Steen[1,2,*]

[1] NORMENT – KG Jebsen Centre for Psychosis Research, Department of Clinical Science, University of Bergen, Bergen, Norway

[2] Dr. Einar Martens Research Group for Biological Psychiatry, Center for Medical Genetics and Molecular Medicine, Haukeland University Hospital, Bergen, Norway

[3] Department of Medical Genetics, Oslo University Hospital, Oslo, Norway

[4] NORMENT – KG Jebsen Centre for Psychosis Research, Department of Clinical Medicine, University of Oslo, Oslo, Norway

[5] Department of Medical Epidemiology and Biostatistics, Karolinska Institutet, Stockholm, Sweden

[6] Center for Psychiatric Genomics, Department of Genetics, University of North Carolina, USA

[7] NORMENT – KG Jebsen Centre for Psychosis Research, Division of Mental Health and Addiction, Oslo University Hospital, Oslo, Norway

* To whom correspondence should be addressed.

Tel: +47 55 97 53 27

Email: vidar.martin.steen@helse-bergen.no

Supplementary Table 1.

Core genes from the lipid biosynthesis and transport list in GSEA analysis of SCZ.

	Gene	RANK IN GENE LIST	RANK METRIC SCORE	RUNNING ES
1	SREBF1	153	7.166	0.1816
2	SREBF2	276	5.432	0.3190
3	ELOVL7	563	4.101	0.4152
4	LRP1	1184	2.930	0.4678
5	GAL3ST1	1638	2.490	0.5154
6	NPC1L1	1979	2.231	0.5606
7	FDFT1	2720	1.859	0.5805
8	INSIG2	3704	1.514	0.5820
9	UGT8	3813	1.484	0.6166

Supplementary Information 2

Simplified graphical design of the study.

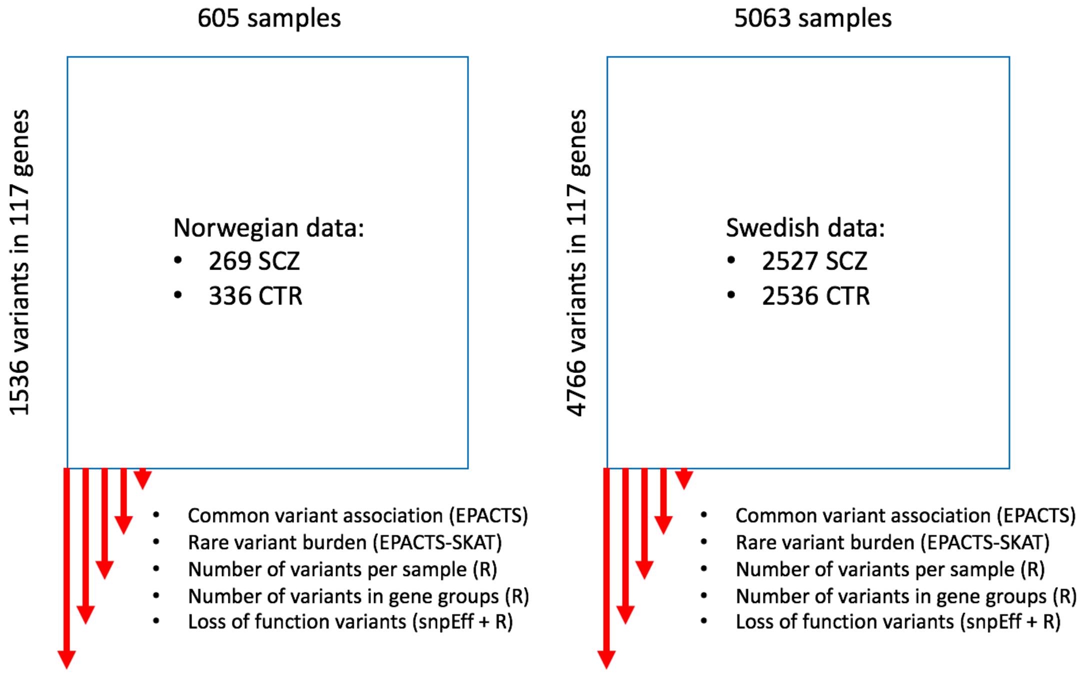

Supplement: Supplementary file 2 [file 41598_2018_25280_MOESM2_ESM.pdf]
